# Multiple Screening of Pesticides Toxicity in Zebrafish and Daphnia Based on Locomotor Activity Alterations

**DOI:** 10.3390/biom10091224

**Published:** 2020-08-23

**Authors:** Akhlaq Hussain, Gilbert Audira, Nemi Malhotra, Boontida Uapipatanakul, Jung-Ren Chen, Yu-Heng Lai, Jong-Chin Huang, Kelvin H.-C. Chen, Hong-Thih Lai, Chung-Der Hsiao

**Affiliations:** 1 Department of Bioscience Technology, Chung Yuan Christian University, Chung-Li 320314, Taiwan; anjumarman390@gmail.com (A.H.); gilbertaudira@yahoo.com (G.A.); 2 Department of Chemistry, Chung Yuan Christian University, Chung-Li 320314, Taiwan; 3 Department of Biomedical Engineering, Chung Yuan Christian University, Chung-Li 320314, Taiwan; nemi.malhotra@gmail.com; 4 Department of Applied Chemistry, Faculty of Science and Technology, Rajamangala University of Technology Thanyaburi, Thanyaburi 12110, Thailand; boontida_u@rmutt.ac.th; 5 Department of Biological Science & Technology, College of Medicine, I-Shou University, Kaohsiung 82445, Taiwan; jrchen@isu.edu.tw; 6 Department of Chemistry, Chinese Culture University, Taipei 11114, Taiwan; lyh21@ulive.pccu.edu.tw; 7 Department of Applied Chemistry, National Pingtung University, Pingtung 900391, Taiwan; hjc@mail.nptu.edu.tw; 8 Department of Aquatic Biosciences, National Chiayi University, 300 University Rd., Chiayi 60004, Taiwan; 9 Center of Nanotechnology, Chung Yuan Christian University, Chung-Li 320314, Taiwan

**Keywords:** zebrafish, daphnia, pesticide, locomotion, behavior, phenomics

## Abstract

Pesticides are widely used to eradicate insects, weed species, and fungi in agriculture. The half-lives of some pesticides are relatively long and may have the dire potential to induce adverse effects when released into the soil, terrestrial and aquatic systems. To assess the potential adverse effects of pesticide pollution in the aquatic environment, zebrafish (*Danio rerio*) and *Daphnia magna* are two excellent animal models because of their transparent bodies, relatively short development processes, and well-established genetic information. Moreover, they are also suitable for performing high-throughput toxicity assays. In this study, we used both zebrafish larvae and water flea daphnia neonates as a model system to explore and compare the potential toxicity by monitoring locomotor activity. Tested animals were exposed to 12 various types of pesticides (three fungicides and 9 insecticides) for 24 h and their corresponding locomotor activities, in terms of distance traveled, burst movement, and rotation were quantified. By adapting principal component analysis (PCA) and hierarchical clustering analysis, we were able to minimize data complexity and compare pesticide toxicity based on locomotor activity for zebrafish and daphnia. Results showed distinct locomotor activity alteration patterns between zebrafish and daphnia towards pesticide exposure. The majority of pesticides tested in this study induced locomotor hypo-activity in daphnia neonates but triggered locomotor hyper-activity in zebrafish larvae. According to our PCA and clustering results, the toxicity for 12 pesticides was grouped into two major groups based on all locomotor activity endpoints collected from both zebrafish and daphnia. In conclusion, all pesticides resulted in swimming alterations in both animal models by either producing hypo-activity, hyperactivity, or other changes in swimming patterns. In addition, zebrafish and daphnia displayed distinct sensitivity and response against different pesticides, and the combinational analysis approach by using a phenomic approach to combine data collected from zebrafish and daphnia provided better resolution for toxicological assessment.

## 1. Introduction

Pesticides are chemically designed to kill multiple insects, weeds, and fungi, and are widely used in agriculture for increasing crop yield and economic income. The rapidly growing human population increases the usage of pesticides to maintain the growing food demands [1]. Pesticides play an important role in agriculture especially for assisting crop quality and high productions [2]. However, many surveys have documented the impact of pesticides and concerns raised on the health risks through various contamination of food and drinking water. Exposure to pesticides may lead to skin irritation, dizziness, nausea, and chronic ailments such as cancer and diabetes [1]. It is, therefore, necessary to understand the chemical structure and formulation of pesticides, the persistence of pesticides, and the characteristics of the affected environment. The aforementioned are all needed to be included in environmental and health risk assessment [3]. 

The excessive use of pesticides leads to undesirable consequences to human health and the environment because of their high biological activity and persistence in the environment. Furthermore, inappropriate handling of pesticides may cause severe acute toxicity [4]. The general population may be exposed to pesticide residues in foods and drinking water [5]. The agrochemical toxicity to non-target aquatic organisms is an important part of chemical ecological risk management [6]. In water bodies, pesticides are exposed to aquatic organisms in mixtures varying in composition over time [7]. However, there has been insufficient knowledge regarding the toxicity of pesticides. 

In the present study, we examined the ecotoxicological effects of 12 different potential pesticides by using neonates of *Daphnia magna* and larvae of zebrafish (*Danio rerio*), which both are excellent aquatic models that have been used extensively in toxicological studies [8]. Previous literature in zebrafish and daphnia had focused on evaluating mortality rate and developmental defects against toxicant exposure in both animal models [2]; however, none of the previous research examined and compared the chemical-induced toxicity at the behavioral level. In this study, we aimed to analyze toxicological response focusing on the behavioral endpoints. *Daphnia magna* is a small and rapidly reproducing aquatic invertebrate organism and has been a well-established model species for ecotoxicity studies. Several studies have reported that daphnids can respond to various factors, such as temperature, photoperiod, food supply, and toxicants [9,10,11]. The toxicity tests of *D. magna* are reliable and *D. magna*-based bioassay is legally adopted in many countries [12]. In the same manner, zebrafish larvae are also widely being used to study the developmental, physiological, and behavior alteration exposed to environmental toxicants [13,14,15]. The larva of zebrafish and daphnia are only a few millimeters long and can be imaged in 6, 12, 24, or 96-well plates. Based on the above-mentioned advantages, zebrafish has been well recognized as an excellent vertebrate model for ecotoxicological research [16,17,18,19]. 

Our objective was to evaluate toxicological information necessary for ecological risk assessment of potential pesticides using various behavioral endpoints. To this end, we conducted experiments of daphnid and zebrafish by following OECD guidelines to determine the potential exposure of pesticides on locomotion activity as mobility and swimming activity are the most sensitive markers widely used in toxicological research as it related to feeding and foraging throughout the life span of the animals [20]. Finally, the locomotor data collected from both zebrafish and daphnia were subject to principal component analysis (PCA) and hierarchical clustering to minimize data complexity and mining potential similarity for behavioral phenomics between zebrafish and daphnia after exposure to diverse type of pesticides.

## 2. Materials and Methods

### 2.1. Zebrafish Larvae Rearing and Maintenance

Zebrafish AB strain stock was obtained from Taiwan Zebrafish Core Facility at Academia Sinica (TZCAS) and kept in the laboratory for toxicity tests. The stock was housed for one month in a system connected with a recirculating aquatic system at 26.5 °C and the conductivity between 300 and 1500 µS with a 10/14 dark-light cycle and maintained pH between 6.8 and 7.5. The fishes were fed two times a day with artemia according to the breeders in an Association for Assessment and Accreditation of Laboratory Animal Care (AAALAC) approved animal facility before the experiment began. One female and two males were netted into a breeding tank for overnight by putting a transparent separator between both sexes to separate them following our previous protocol [21,22]. The next morning, the barrier was removed. After embryos were collected and sanitized, they were placed at 28 °C in methylene blue water. On the following day, all of the dead embryos were removed and the E3 medium was changed every day until day three of post-fertilization. Zebrafish experimental protocol and ethics were approved by the Chung Yuan Christian University animal care and welfare committee (Number: CYCU109001, issue date 20 January 2020).

### 2.2. Daphnia Rearing and Maintenance

*Daphnia magna* stock was obtained from Freshwater Bioresource Center at National Chiayi University and kept in the laboratory for toxicity tests. A single clone of tested daphnia was obtained by parthenogenetic reproduction and maintained in 12 L of culture medium under a light-dark period of 14 h: 10 h with a temperature of 20 ± 2 °C and pH 6.7 and a water conductivity between 300 and 1500 µS. The animals were fed by either green alga or beaker yeast suspension and the cultured water was exchanged every two days (≥80%) [4]. Locomotion activity of daphnia is depended upon the body sizes; therefore, a filter was used to make sure the uniform body size. After the separation, we chose the medium size (0.6–1 mm) of daphnia in our experiment and incubated around 50 daphnias in a petri dish for each treatment group. 

### 2.3. Chemical Exposure

**Figure 1:**
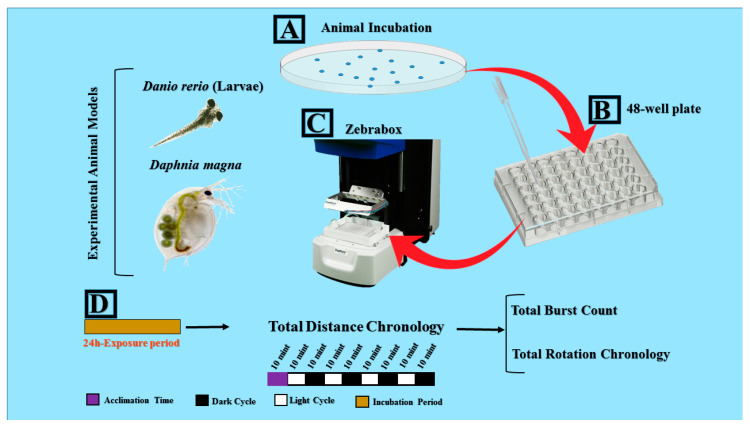
Locomotor toxicity assessment of pesticides by using both water flea (*Daphnia magna)* neonates and zebrafish (*Danio rerio*) larvae. (**A**) Experimental animals were incubated in pesticide-containing solutions for ~24 h. (**B**) Experimental animals were then transferred to 48-well plate with the aid of a pipette. (**C**) ZebraBox behavioral observation instrument machine was used to perform high-throughput locomotor toxicity assays. (**D**) Experimental design and endpoints such as total distance traveled, total burst, and rotation movement, and alternating light/dark transition are represented.

Pesticides of ≥98% purity were purchased from Aladdin Bio-Chem Technology Co., LTD Shanghai, and dissolved in 0.01% organic solution Acetone. Later, they were diluted to 1 ppb for determining the swimming behavior of the animal models. Afterward, a group of 30 zebrafish larvae aged at 96 hpf and a group of 50 daphnias of size ranging from 0.6–1 mm were randomly netted and chosen for both control and treatment groups. The sample size of zebrafish applied in the current experiment was based on several prior studies [19,20,23]. Meanwhile, we used 48 *D. magna* for each treatment group. The determination of this sample number was based on the previous study in other invertebrates, which were *Macrobrachium ronsebergii* and *Marcrobrachium carcinus* [20]. Each group was placed in a single 9 cm petri dish with either 50 mL of normal water (in case of control) or 50 mL of 1 ppb pesticide solution (in case of treatment group) for ~24 h for both species according to the prior study [24]. After ~24 h incubation, the control and pesticide-exposed groups of both species were individually transferred to a 48-well plate for locomotor activity measurement. The acclimation time is necessary for an individual especially when they were being transferred to the novel environment. Next, after they were transferred into the 48-well plate, we gave approximately 1 h of acclimation time before they were placed on the plate of the ZebraBox (ViewPoint Life Sciences, Inc., Civrieux, France) machine. After the 48-well plate was placed into the ZebraBox, we gave further 10 min of acclimation time before starting the recording process as shown in Figure 1. In Table 1, we summarized the name, functional grouping, environmental concentration, and acute toxicity level of 12 pesticides (three fungicides and 9 insecticides) used to perform toxicity assessment in both zebrafish and daphnia. The concentration of all pesticides used in this study was 1 ppb, which is 1000 folds less than EC50 reported in crustaceans by WHO (see summary in Table 1). The overview of the experiment can be found in Figure 1.

### 2.4. Automated Imaging of Swimming Behaviors Analysis

For this experiment, we used an automated high throughput imaging system developed by Viewpoint Company (ViewPoint, 3.22.3.85, ViewPoint Life Sciences, Inc.: Civrieux, France, 2014, http://www.viewpointlifesciences.com). Infrared illumination is used in the Viewpoint system for imaging in the dark. Video tracking analyses were performed into 48-well plates and tracked individually by putting one individual in one well contain 800 µL of exposure medium. The video was recorded for 80 min for both animal models in response to 10 min interval of light and dark transitions during the daytime from 11:00 to 16:00. Later, we used the same video for burst count and rotation movement. The activity of each zebrafish larvae and daphnia were measured every minute of recording time for all of the three endpoints. In the case of zebrafish larvae, our sample number was 24, thus, we divided the 48-well plates into two parts for testing two different compounds together. Locomotion activity was examined by deriving three main endpoints, namely, total distance traveled, burst count, and rotation count. According to the previous research, the movement was categorized into cruising (commonly measured normal speed), large movement (short, powerful, and intermittent activity), and inactivity (freezing movement) [23]. We classified the corresponding velocities with greater than 20 mm/s, 0.5 to 20 mm/s, and less than 0.5 mm/s as large movement, cruising movement, and inactivity, respectively. Furthermore, for the burst activity count, the quantization of video track parameters was set as burst, 20; freeze (no movement) 2; detection threshold, 20. In addition, we also evaluated the rotation count that was considered as one of the sensitive markers for locomotor activity by changing the angle of the body part in response to different environmental stimuli such as food, light, toxicants, and the fear of predators [31]. Here, we determined the clockwise and counter-clockwise movements of zebrafish larvae in the unit of a millimeter and considered a diameter greater than 2 mm as one rotation and rotation below 2 mm was neglected. In addition, the back angle was set at 60°.

### 2.5. Statistical Analysis

All of the data were expressed as the mean with SEM (standard error of the mean) to display more representative data. All statistical tests were conducted either through Brown-Forthsythe and Welch ANOVA (analysis of variance) test, Two-way ANOVA test continued uncorrected Fisher’s LSD (least significant difference) test, or Mann-Whitney test to compare the treatment groups with the control group to observe the pesticide effects. The Brown-Forthsythe and Welch ANOVA test was applied since we assumed that the groups had unequal variances. This assumption was made because the coefficient of variation for each group was calculated prior to the Brown-Forthsythe and Welch ANOVA test. Afterward, it was found that all of the groups exhibited a high percentage of the coefficient of variance, which was at least more than 30% for zebrafish and 100% for daphnia, and in the laboratory studies, it is expected to have CV (coefficient variation) less than 10% [32]. Furthermore, the two-way ANOVA with uncorrected Fisher’s LSD was used to compare the light and dark mean throughout the 80 min of the experiment. In the two-way ANOVA test result, the row factor was the time while the column factor was the treatment. Lastly, the Mann-Whitney test was conducted to calculate the differences of all behavioral endpoints between light and dark cycles in every group. Statistical tests were performed using GraphPad Prism (GraphPad Prism, 8.0.2, GraphPad Software, Inc.: San Diego, CA, USA, 2019, https://www.graphpad.com/). The statistical significances were displayed as “ns” for no statistical significance, “*” for *p* < 0.05, “**” for *p* < 0.01, “***” for *p* < 0.001, and “****” for *p* < 0.0001).

### 2.6. Principal Component Analysis (PCA), Heatmap, and Hierarchical Clustering Analysis

All of the behavior endpoints were pre-processed to obtain the average value and the differences from the average of the control were calculated and inserted into an excel file using Microsoft Excel. Afterward, the excel file was converted into a comma-separated values type file (.csv). Prior to the PCA analysis, all of the raw data were normalized to their control to minimize the variation between the data from zebrafish larvae and *D. magna*. After normalization, clustering was then performed across all of the behavioral endpoints by using open access ClustVis online tool (https://biit.cs.ut.ee/clustvis/). Unit variance scaling for each row was applied to treat each variable equally. In addition, singular value decomposition (SVD) with the imputation method was used to calculate the principal component since there were no missing values in the dataset. 

## 3. Results

### 3.1. Total Distance Chronology for Zebrafish after Pesticide Exposure

**Figure 2:**
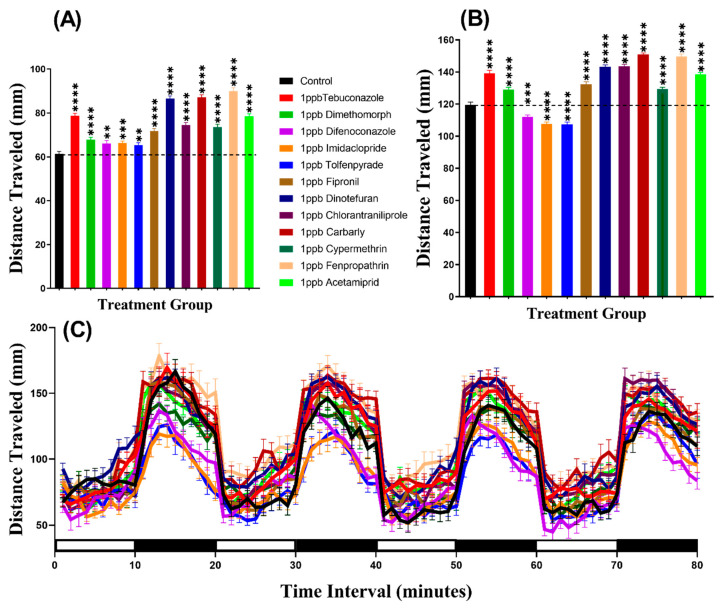
Average distances of zebrafish larvae traveled in the control and 24-h 1 ppb pesticide treatments. (**A**) Total traveled distance during the light cycle. (**B**) Total traveled distance during the dark cycle. (**C**) The pattern of locomotor activity during light/dark transitions. The data were expressed as the Mean ± SEM and analyzed by Two-way ANOVA and Brown–Forthsythe and Welch ANOVA test. Dunnett’s multiple comparison test was carried out for comparing all treatments with control to obtain the pesticide effects (*n* = 24; ** *p* < 0.01, *** *p* < 0.001, **** *p* < 0.0001).

**Figure A1:**
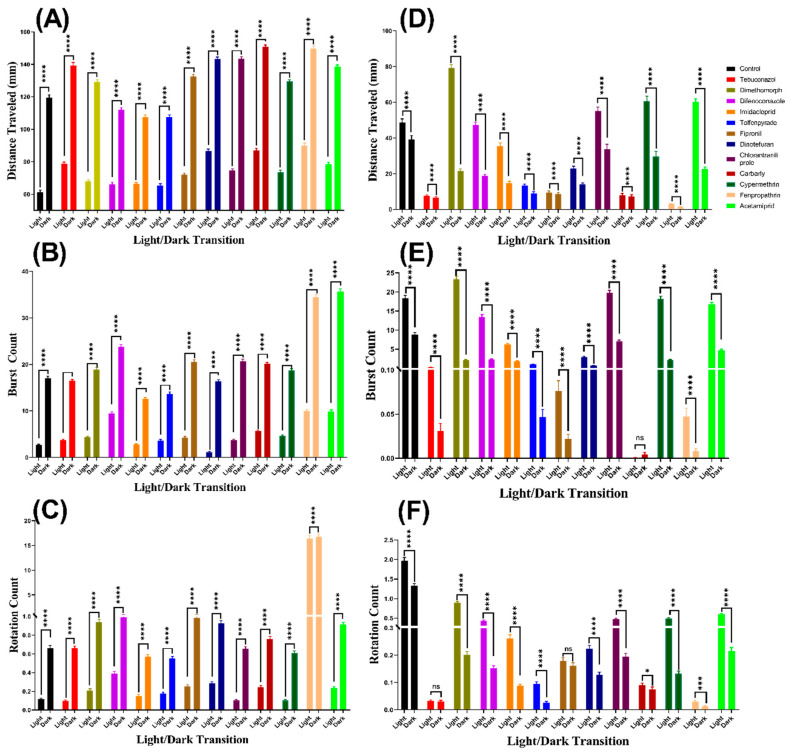
Comparison of (**A**,**D**) distance traveled, (**B**,**E**) burst count, and (**C**,**F**) rotation count between the light and dark cycles in each group of (**A**–**C**) zebrafish and (**D**–**F**) *Daphnia magna*, respectively. The data are expressed as the Mean ± SEM and analyzed by Mann–Whitney test (*n* zebrafish = 24, *n Daphnia magna* = 48; ns = not significant, * *p* < 0.05, *** *p* < 0.001, **** *p* < 0.0001

In the light period, we found that all pesticides induced hyperactivity. Brown-Forthsythe and Welch test revealed hyperactivity for the fish incubated in either Tebuconazole (*p* < 0.0001), Dimethomorph (*p* < 0.0001), Difenoconazole (*p* = 0.0068), Tolfenpyrade (*p* = 0.0059), Imidacloprid (*p* = 0.0006), Fipronil (*p* < 0.0001), Dinotefuran (*p* < 0.0001), Chlorantraniliprole (*p* < 0.0001), Carbarly (*p* < 0.0001), Cypermethrin (*p* < 0.0001), Fenpropathrin (*p* < 0.0001), or Acetamiprid (*p* < 0.0001) (Figure 2A). Meanwhile, in the dark period, all pesticides caused hyperactivity in zebrafish larvae with *p* < 0.0001 except Difenoconazole (*p* = 0.0003), Imidacloprid (*p* < 0.0001), and Tolfenpyrade (*p* < 0.0001) which were responsible for hypoactivity (Figure 2B). Furthermore, we performed Two-way ANOVA to compare the locomotor activity throughout the 80 min of the light and dark phases and we found that the dimethomorph-treated zebrafish larvae exhibited a similar pattern of distance traveled with the control group, while the other groups displayed different patterns (Table A2). In addition, regarding the locomotor activity differences between the light and dark cycles, the result indicated that all of the zebrafish groups displayed higher locomotor activity in the dark period than in the light period (Figure 2C, Figure A1) which is consistent with previously published reports [33,34]

### 3.2. Burst Count Movement for Zebrafish after Pesticide Exposure

**Figure 3:**
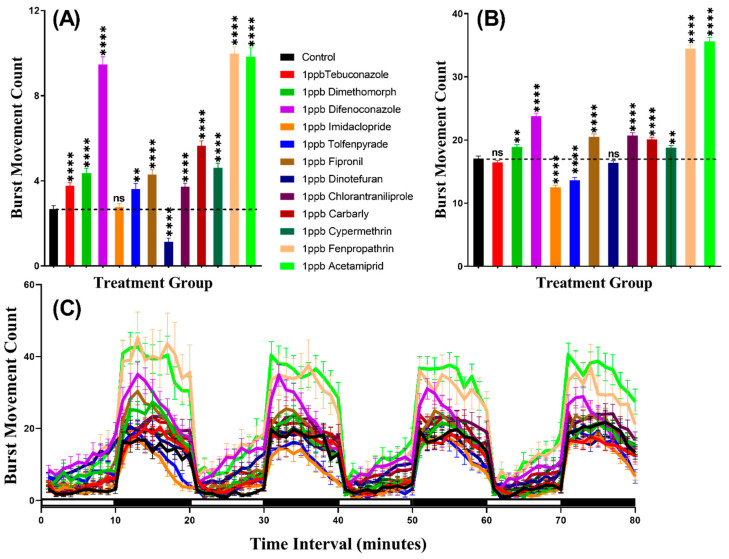
Total burst count of control and 1 ppb pesticides treated zebrafish larvae after a 24-h exposure. (**A**) The total number of bursts observed during the light cycle. (**B**) The total number of bursts observed during the dark cycle. (**C**) The pattern of burst count during light/dark transition. The data are expressed as the Mean ± SEM and they were analyzed by Two-way and Brown–Forthsythe and Welch ANOVA test. Dunnett’s multiple comparison test for comparing all treatments with control was carried out to observe the pesticide effects (*n* = 24; ns = not significant, ** *p* < 0.01, **** *p* < 0.0001).

We considered burst count as the rapid movement during the experiment by counting the animal body movement with more than 20 pixels per second, which helps to evaluate the locomotion activity. In agreement with the total distance result, this result indicated that zebrafish displayed more burst movement in the dark period than in the light period. Furthermore, the burst counts of zebrafish larvae exposed to pesticides showed hypoactivity against Dinotefuran (*p* < 0.0001). In contrast, zebrafish larvae showed locomotion hyperactivity against all other pesticides except Imidacloprid which had not significantly deviate from the control group (*p* = 0.7297). In the dark cycle, larvae incubated in Tebuconazole and Dinotefuran had not significantly different burst count, however, Dimethomorph (*p* = 0.0014), Difenoconazole (*p* < 0.0001), Fipronil (*p* < 0.0001), Chlorantraniliprole (*p* < 0.0001), Carbaryl (*p* < 0.0001), Cypermethrin (*p* = 0.0020), Fenpropathrin (*p* < 0.0001) and Acetamiprid (*p* < 0.0001) resulted elevation of burst count as compared to control group. Meanwhile, Imidacloprid (*p* < 0.0001) and Tolfenpyrade (*p* = 0.0001) were responsible for decreased burst count in the dark cycle (Figure 3A,B). Furthermore, Two-way ANOVA results showed that all of the treatment groups, except the tebuconazole group, displayed significantly different patterns of burst movement count compared to the control group throughout the 80 min of the light and dark phases (Table A2). In addition, the burst movement count in the whole light and dark periods during the experimental period in each group were observed and the results revealed that zebrafish responded to the light cycle more clearly, which was similar to the total distance result (Figure 3C, Figure A1). 

### 3.3. Swimming Orientation for Zebrafish after Pesticide Exposure

**Figure 4:**
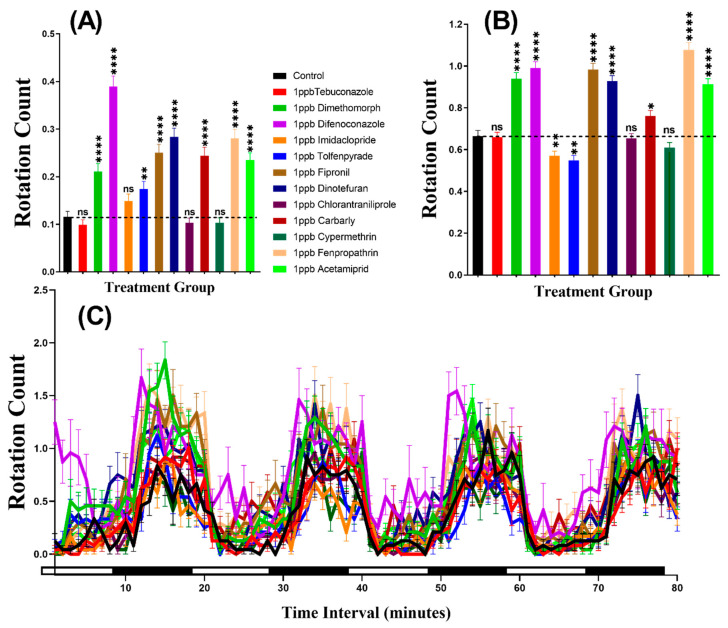
Total rotation count of zebrafish larvae in the control and 1 ppb pesticide treatment after 24-h exposure. (**A**) Total rotation count during the light cycle. (**B**) Total rotation count during the dark cycle. (**C**) The pattern of rotation count during light/dark transition. The data are expressed as the Mean ± SEM and they were analyzed by Two-way ANOVA and Brown–Forthsythe and Welch ANOVA test. Dunnett’s multiple comparison test for comparing all treatments with control was carried out to observe the column (pesticide) effects (*n* = 24; ns = not significant, * *p* < 0.05, ** *p* < 0.01, **** *p* < 0.0001).

The swimming orientation (clockwise or counter-clockwise) varies in the response to different factors such as food, light, or predator pressure. In this study, the result indicated that zebrafish displayed more rotation movement in the dark period than in the light period in all of the groups. Furthermore, our statistical analysis revealed higher rotation count in group treated with Dimethomorph (*p* < 0.0001), Difenoconazole (*p* < 0.0001), Tolfenpyrade (*p* = 0.0035), Fipronil (*p* < 0.0001), Dinotefuran (*p* < 0.0001), Carbaryl (*p* < 0.0001), Fenpropathrin (*p* < 0.0001), and Acetamiprid (*p* < 0.0001) than in control animals during the light cycle. However, compounds such as Tebuconazole, Imidacloprid, Chlorantraniliprole, and Cypermethrin behaved at control levels (Figure 4A). In the same manner, most of the pesticides resulted very clear rotational movement elevation during the dark period (Figure 4B). Statistical results revealed that compound such as Dimethomorph (*p* < 0.0001), Difenoconazole (*p* < 0.0001), Fipronil (*p* < 0.0001), Dinotefuran (*p* < 0.0001), Chlorantraniliprole (*p* = 0.0100), Fenpropathrin (*p* < 0.0001), and Acetamiprid (*p* < 0.0001) marked higher rotation movement in dark cycle. Meanwhile, the other two compounds, which are Imidacloprid (*p* = 0.0074) and Tolfenpyrade (*p* = 0.0011), showed lower rotation count while three other compounds Tebuconazole (*p* = 0.8851), Chlorantraniliprole (*p* = 0.7513) and Cypermethrin (*p* = 0.1306) showed no significant difference as compared to control. Furthermore, Two-way ANOVA was also performed to compare the rotation count throughout the 80 min of the light and dark phases and it was found that the imidacloprid, tolfenpyrade, chlorantraniliprole, and cypermethrin-treated zebrafish larvae exhibited a similar pattern of rotation count with the control group, which was not shown in other treated groups (Table A2). In addition, we also compared the rotation count in each cycle to estimate the movement orientation differences in every group. Later, it was found that the rotation count responded to the light than dark, but not as clear as the other two endpoints (Figure 4C, Figure A1). 

### 3.4. Total Distance for Daphnia Magna after Pesticide Exposure

**Figure 5:**
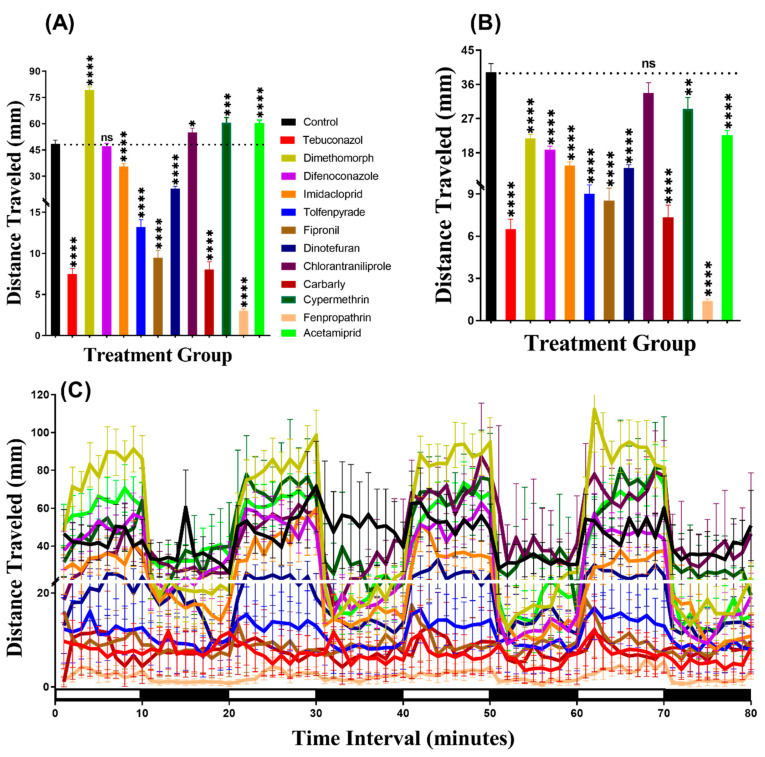
The distances traveled in the control and 1 ppb pesticide treatments of *Daphnia magna* after a 24-h exposure. (**A**) Total distance traveled during the light cycle. (**B**) Total distance traveled during the dark cycle. (**C**) The pattern of locomotion activity during light/dark transition. The data are expressed as the Mean ± SEM and they were analyzed by Two-way ANOVA and Brown–Forsythe test. Dunnett’s multiple comparison test for comparing all treatments with control was carried out to determine the pesticides effects (*n* = 48; ns = not significant, * *p* < 0.05, ** *p* < 0.01, *** *p* < 0.001, **** *p* < 0.0001).

The swimming velocity and distance traveled of *D. magna* are depended on its size [35]. Animals with similar size swim at a similar velocity, which is independent of their ages [35]. Therefore, we chosen daphnia with a similar size for both experimental and control groups to measure the total distance traveled, burst, and rotation movement chronology as performed in the zebrafish analyses by using ZebraBox. In contrast to the zebrafish larvae, the results showed that *D. magna* was more active in the light period than in the dark period and covered maximum distance traveled (Figure A1). This result indicated daphnia displayed more intense locomotor activity in the light period than in the dark period, which is distinct to the pattern display by zebrafish. The higher locomotor activity in the dark period than in the light period for daphnia is consistent with the previously published report [36]. Meanwhile, in the light period, Difenoconazole had no significant effects (*p* = 0.6229) on daphnia locomotion activity. Compounds such as Dimethomorph (*p* < 0.0001), Chlorantraniliprole (*p* = 0.0440), Cypermethrin (*p* = 0.0006), and Acetamiprid (*p* < 0.0001) significantly boosted activity as compared to the control counterpart (Figure 5A). However, all other pesticides like Tebuconazole, Imidacloprid, Tolfenpyrade, Fipronil, Dinotefuran, Carbaryl, and Fenpropathrin reduced locomotor activity in the light period (*p* < 0.0001) (Figure 5A). In the dark period, except for Chlorantraniliprole (*p* = 0.1169), the majority of the tested compounds significantly reduced the locomotor activity in *D. magna* (Figure 5B). Furthermore, we also conducted the Two-way ANOVA to compare the locomotor activity throughout the 80 min of the light and dark phases. Later, while the other groups displayed different patterns, we found that the chlorantraniliprole and cypermethrin-treated daphnia exhibited a similar pattern of distance traveled with the control group (Table A2). In addition, Figure 5C showed the locomotion activity during both and light/dark transition for daphnia. The pattern of response to light and dark transition of water flea was not so distinct and clear as observed in zebrafish larvae (Figure 5C).

### 3.5. Burst Count Movement for Daphnia magna after Pesticide Exposure

**Figure 6:**
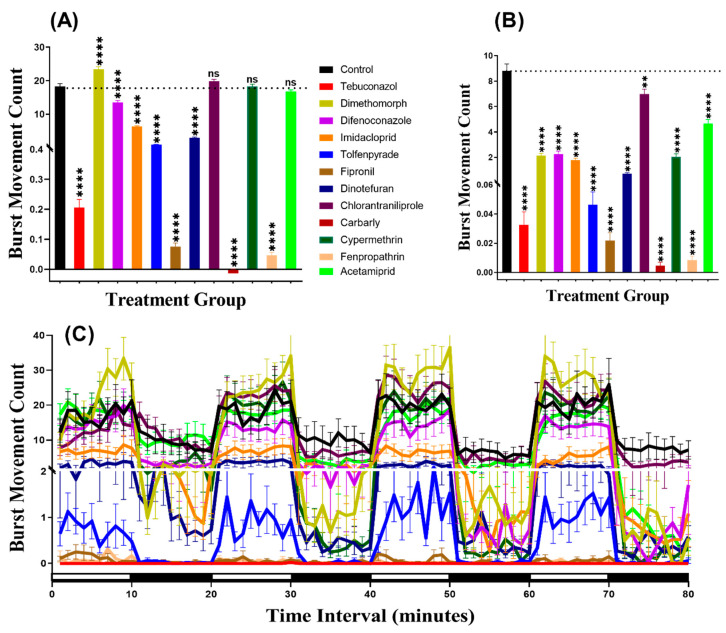
The total burst count of *Daphnia magna* in the control and 1 ppb pesticide after a 24-h exposure. (**A**) Total burst count during the light cycle. (**B**) The total number of bursts during the dark cycle. (**C**) Burst count pattern during the light/dark transition. The data are expressed as the Mean ± SEM and analyzed by Two-way ANOVA and Brown–Forsythe test. Dunnett’s multiple comparison test for comparing all treatments with control was carried out to observe the pesticides effects (*n* = 48; ns = not significant, ** *p* < 0.01, **** *p* < 0.0001).

The rapid simultaneous movement of *D. magna* was calculated after being exposed to pesticides. The same video recorded for the total distance was used to evaluate the burst movement. Burst is one of the significant effects in locomotion activity to evaluate swimming irregularity and anxiety in zebrafish larvae [31]. The results indicated that daphnia displayed more burst movement in the light period than in the dark period. Furthermore, during the light cycle, the daphnia treated with Chlorantraniliprole (*p* = 0.1509), Cypermethrin (*p* = 0.9674), and Acetamiprid (*p* = 0.1117) had similar burst count as their control counterpart. However, a group of daphnia incubated in Dimethomorph showed a higher burst movement (*p* < 0.0001), while the other pesticides resulted in lower burst counts (Figure 6A). In contrast, during the dark period of light/dark transition, all the pesticides resulted in a decrease (*p* < 0.0001) in the burst counts (Figure 6B). Furthermore, to compare the locomotor activity throughout the 80 min of the light and dark phases, Two-way ANOVA was carried out and the result showed that the dimethomorph and chlorantraniliprole-treated daphnia exhibited a similar pattern of burst movement count with the control group, while this phenomenon was not observed in the other treatment groups (Table A2). In addition, the pattern of the response of the animal to light/dark transition showed that the peaks of burst count rise at light and fall sharply during the dark transition in all of the groups, except in the carbaryl-treated group (Figure 6C, Figure A1). Taken together, we concluded the burst movement in daphnia is a better and more sensitive endpoint than the total distance traveled to evaluate the adverse effect triggered by pesticide exposure.

### 3.6. Swimming Orientation for Daphnia magna after Pesticide Exposure

**Figure 7:**
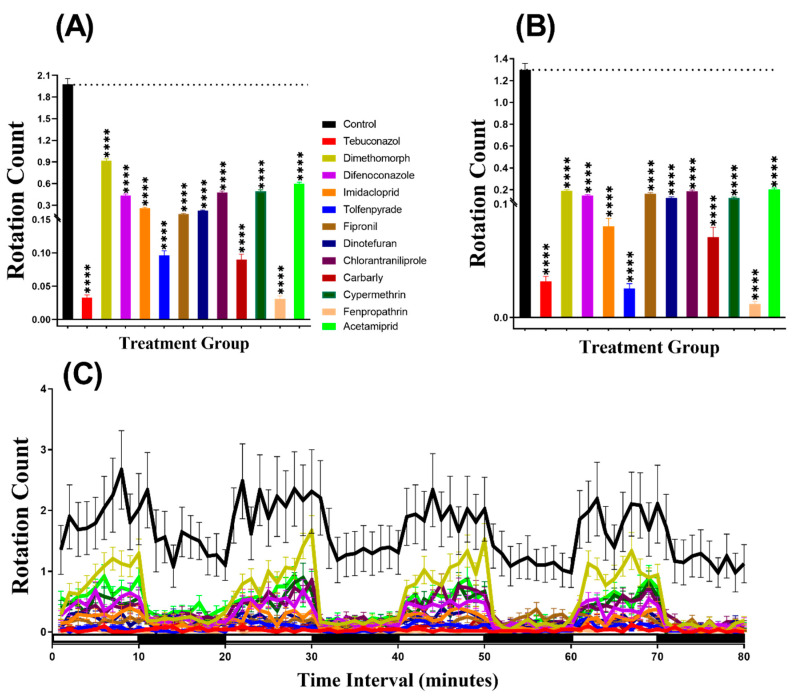
Total rotation count of control and *Daphnia magna* after 24-h exposure to 1 ppb pesticides. (**A**) Rotation counts during the light cycle. (**B**) Rotation count during the dark cycle. (**C**) The pattern of rotation counts in light and dark transition. The data are expressed as the Mean ± SEM and they were analyzed by Two-way ANOVA and Brown–Forsythe test. Dunnett’s multiple comparison test for comparing all treatments with control was carried out to observe the effect of the pesticides (*n* = 48; **** *p* < 0.0001).

The swimming orientation (clockwise and counterclockwise) of *D. magna* treated with pesticides were analyzed by using the video recorded for swimming activity. From the result, the rotation count of daphnia in the pesticide-treated groups was noticed for its consistent lower in both light and dark periods (Figure 7A,B). In addition, we also found that the rotation movement in Daphnia is a very sensitive behavior endpoint since this endpoint can be intensely be affected by low dose pesticide treatment with the lowest *p*-value (*p* < 0.0001). Furthermore, Two-way ANOVA was also performed to compare the rotation count throughout the 80 min of the light and dark phases and we found that all of the treated daphnia groups exhibited a significantly different pattern of rotation count with the control group (Table A2). In addition, regarding the movement orientation differences between the light and dark cycles, the result showed that daphnia displayed more rotation movement in the light period than in the dark period, except for tebuconazole, fipronil, and fenpropathrin-treated groups (Figure 7C, Figure A1).

### 3.7. PCA Analysis and Clustering of Phenomics Data

**Figure 8:**
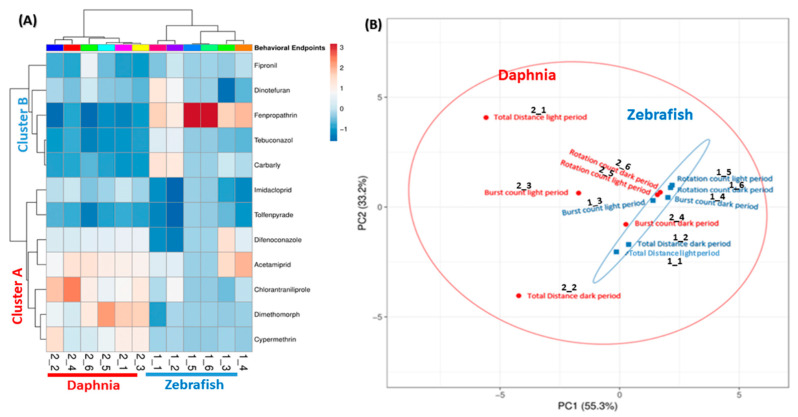
Hierarchical clustering for locomotor activity changes collected from both zebrafish and daphnia based on the pesticide dimension. (**A**) Heat map color key. Color in the heat map represents the deviation from the average of the control: red, higher activity; light blue, lower activity. (**B**) Principal component analysis (PCA) showing the group of pesticides cluster together based on their response to behavior. Unit variance scaling is applied to rows; singular value decomposition (SVD) with imputation is used to calculate principal components. X and Y axis show principal component 1 and principal component 2 that explains 55.3% and 33.2% of the total variance, respectively.

Clustering is a statistical technique by grouping a set of data in a way that similar data are considered the same group, which is important to organize, classify, and summarize the data. It also gives us an idea about how diverse cluster groups are. Here, we analyzed 12 pesticides, using six different endpoints in two different animal models species. Thus, our data had multiple variants because of the multiple endpoints, thus, we took help from this statistical technique to reduce data complexity. In the present study, two clusters were made; one clustering based on behavioral endpoints, and another clustering is based on the activity of the pesticide. Based on the behavior, there were two major clusters obtained, one pertains *D. magna* (highlighted by red color) and the other was *D. rerio* (highlighted by blue color). None of the behavior endpoints of D. *magna* and zebrafish was clustered together. Both species had different locomotor activity patterns after exposure to diverse types of pesticides. This phenomenon was reasonable since both of the species responded differently to the light and dark stimuli with zebrafish larvae became active in the dark period while *D. magna* was more active in the light period of time (Figure 8A). In addition, the variable correlation plots were also consistent with the heat map clustering for behavioral endpoints. This result was shown by all behavioral endpoints of zebrafish larvae and daphnia that were far from each other and grouped separately (Figure 8B). Furthermore, from Figure 8B we concluded zebrafish behavioral endpoints were more consistent and reliable as compared to daphnia. All the behavior endpoints in the case of daphnia are far from each other, thus, they were making a big circle (showing in red color). Meanwhile, in the zebrafish, all of the behavior endpoints were nicely compact and grouped very close to each other and making the small eclipse (showing in blue color). Moreover, the results show that the 12 tested pesticides were clustered in two major groups cluster A and B. The cluster A contained Acetamiprid, Dimethomorph, Chlorantraniliprole, Tolfenpyrade, Difenoconazole, Imidacloprid, and Cypermethrin while the cluster B consisted of the other five pesticides, including Fenpropathrin, Dinotefuran, Carbaryl, Tebuconazole, and Fipronil. 

## 4. Discussion

The idea of inter-species comparison by using both fish and water flea models in ecotoxicological fields has been proposed and several studies demonstrated that the sensitivities to various compounds are similar within the different genus [1,2,3]. Previous research focuses on mortality rates and developmental defects for daphnia and zebrafish interspecies comparison has been well addressed, but none of the literature targeted the question based on locomotor activity approach by using multi toxicants and animal models [1,2,3]. The summary of some prior studies that used different model species for toxicity test can be found in Table A1. As it is already well known, a behavioral response is well recognized as a more intense and sensitive endpoint than mortality and teratology for toxicological studies [37,38,39,40,41]. For example, daphnia behavioral changes have been selected as a model for the early warning of aquatic organophosphorus pesticide contamination by the on-line monitoring approach [41]. Furthermore, behavioral endpoints indicate that animals interact with biotic and abiotic factors of the environment that responded to various internal and external stimuli that are important for animal survival. 

Living organisms demonstrate diverse behaviors, which are usually difficult to quantify and compare. With the aid of a high-throughput behavioral tracking system as well as an omics analysis tool kit, in this study, we were able to conduct multiple dimensional assays to explore the locomotor activity alterations in both daphnia and zebrafish for the first time. Clustering analyses have been recommended for multifactorial and multi-variated data set such as transcriptomics and gene expression [42,43]. In the previous zebrafish study, the clustering approach was also used to digest the high dimensional behavioral data with neuroactive molecules for separating the compounds with a similar function [44]. In the current study, the most significant improvement and achievement were that we adopted an Omic tool kit to address the locomotor activity alteration by using three major endpoints in two aquatic animal models species after exposed to 12 pesticides. Three endpoints, which were total distance traveled, burst, and rotation counts were selected, measured, and compared. The first endpoint describes the total swimming activity from immobility to high-speed movement. The second and third endpoints recorded the swimming patterns of experimental animals. Interestingly, even though at a very low dose, all of the pesticides used in this study were still able to trigger vigorous behavioral alterations by producing either hyper- or hypo-locomotor activity in both zebrafish and daphnia. However, we found it was difficult to evaluate pesticide toxicity if only based on locomotor endpoints since some pesticides triggered locomotor activity responses at different magnitudes in either zebrafish or daphnia. For example, some pesticides induced locomotor immobilization in one species but altered swimming patterns in another. Thus, this phenomenon suggested that the action mechanisms for specific pesticides might be different in the tested target animals. Furthermore, these different activities might due to the diverse chemical structure and function of the tested chemicals [3,45]. In addition, this is the reason why some pesticides produced locomotor hyperactivity in zebrafish, but induced hypoactivity in water flea. 

To overcome this species-specific problem, we combined data collected from both zebrafish and daphnia to generate a higher dimensional dataset, and later, hierarchical clustering and PCA analyses were used to reduce data complexity and to build behavioral similarity relationship. By side-by-side comparison, we found zebrafish and *D. magna* displayed distinct and complementary responses toward the light/dark transition. The zebrafish larvae were active in the dark period while daphnia was active in the light period. In zebrafish larvae, it is already well-known that they display a startle response when exposed to a sudden change in light intensity [46]. This visual startle may presage an escape response that would normally be evoked by a looming predator [47]. Meanwhile, the photo-dependent swimming response observed in the daphnia might be linked to the circadian rhythm and whether animals are diurnal or nocturnal would need further exploration. In addition, we found that there was a tendency in both species that all of the endpoints were related to each other. For example, when the total distance traveled endpoint value of one treated group was significantly higher than the control group, there was a high probability that the other endpoint values were also found to be significantly higher than the control group in the same manner. 

However, more relevant to our goal, most of the pesticides induced hyperactivity in zebrafish, which was shown in all endpoints, while in the case of daphnia, many pesticides produced hypoactivity. Furthermore, the locomotor activity of zebrafish was consistent in both light and dark cycles. For example, if zebrafish larvae showed hyperactivity against one pesticide in light, mostly, it also showed hyperactivity in dark as well through all endpoints. However, in daphnia, some compounds produced hyperactivity in the light cycle but no hyperactivity was noted in the dark period against any single compound. This inhibited or hyperactive behavior could explain both zebrafish and *D. magna* had impaired tendencies to change swimming directions and cognitive ability. This result might also suggest that the mechanism of pesticide action could be different for both species.

The pesticide used in this study is originally designed to target insect endogenous proteins. In previous studies, some insects like Mediterranean flour moth and *Drosophila melanogaster* [3] have been used as a model for pesticide toxicity assessment. Meanwhile, daphnia is an aquatic Crustacean species, which is phylogenetically close to insects than zebrafish. Therefore, daphnia has been recognized as a good aquatic invertebrate model for insecticide/pesticide toxicity assessment [25,48,49]. In this study, we provided data to support this idea. From the results, daphnia displayed more dynamic alteration for their locomotor behavioral alterations in both light and dark periods than their zebrafish counterpart. Based on the PCA, it is intriguing to find behavioral variation patterns between both species were distinct from each other when they are exposed to diverse types of pesticides (Figure 8B) suggesting different mechanisms of action. The locomotor response of daphnia was more dynamic as it altered in the presence and absence of light suggesting daphnia is more sensitive than zebrafish embryos especially for metals, pesticides, nitrogen compounds, and solvents as stated in the prior study [50]. Therefore, daphnia can be used as the initial screening invertebrate model and zebrafish can be used as follow up vertebrate models due to their different sensitivity toward chemical pollutions. In addition, this sequential toxicity assay idea has been proposed by Jang and colleagues [8] by demonstrating that heterogeneous substances toxicity can be first screened by daphnia to find the optimal concentration range and tested in zebrafish to explore its potential organ-specific toxicity afterward. Our study also supported this idea that the utilization of both daphnia and zebrafish can provide more insight toward chemical toxicity. 

## 5. Conclusions

This study elaborated on an approach to analyzing the locomotor activity alterations of two common models of an invertebrate (water flea) and vertebrate (zebrafish) in response to 12 different pesticides exposure for the first time. The data showed that exposure to diverse types of pesticides could trigger either hypo- or hyper-locomotor activities in different animal models. The behavioral endpoints of zebrafish after pesticide exposure displayed in more relevant and consistent ways compared to the water flea. Water flea, on the contrary, displayed a more strong and sensitive response than zebrafish against pesticide exposure. Among three behavior endpoints, total burst counts identified as a more sensitive behavioral marker for toxicological analysis. Furthermore, zebrafish and daphnia displayed distinct sensitivity and response against different pesticides, and the combinational analysis by using a phenomic approach to combine data collected from Zebrafish and daphnia provided a better resolution for toxicological assessment. 

## Figures and Tables

**Table 1:** Information for the twelve pesticides used to perform locomotor activity assay in both zebrafish and daphnia studies.

Number	Pesticide	Functional Grouping to	WHO GHS Classification for Aquatic Acute Toxicity *	Aquatic Environmental Concentration µg/L	Reference
1	Tebuconazole	Triazole fungicide	1	N.A.	N.A.
2	Difenoconazole	Triazole fungicide	1	9.1	[25]
3	Dimethomorph	Morpholine fungicide	N.A.	24.40	EPA, 1998
4	Imidacloprid	Neonicotinoid insecticide	1	320	[26]
5	Tolfenpyrad	Pyrazole insecticide	1	26.9	EPA, 2014
6	Fipronil	Phenylpyrazole insecticide	1	0.117	[27]
7	Dinotefuran	Neonicotinoid insecticide	N.A.	9.64	US EPA, 2004
8	Chlorantraniliprole	Ryanoid insecticide	1	N.A.	N.A.
9	Carbaryl	Carbamate insecticide	1	0.125	[28]
10	Cypermethrin	Synthetic pyrethroid insecticide	1	3.5	[29]
11	Fenpropathrin	Pyrethroid insecticide	1	undetectable	[30]
12	Acetamiprid	Neonicotinoid insecticide	N.A.	0.0544	[30]

* WHO GHS acute aquatic toxicity definition is under 96 h post-treatment, LC50, and less than 1 ppm for fish or after 48 h treatment, EC50, and less than 1ppm for Crustaceans. N.A. not available.

## Appendix A

**Table A1:** Summary of papers that have used different model species for toxicity test dealing with different endpoints.

Chemicals Tested	Concentration	Endpoints Tested	Main Highlights	References
Ionic and Nanosilver	10, 20, 30, and 50 nM.	Mortality and growth	Toxicity increased with decreasing particle size. LC50 for nanosilver was 10 to 50 times greater than Ag+ (based on mass concentration).	[51]
R-Metalaxyl and rac-Metalaxyl in Acute, Chronic, and Sublethal condition	R-metalaxyl: 0.5, 16, and 169 mg/L for algae, *Daphnia magna* and zebrafish respectively. rac-metalaxyl: 0.05, 300, and 170 mg/L for algae, *Daphnia magna* and zebrafish respectively.	LC50 for 24, 48, and 96 h. Na,K-ATPase enzyme activity in adult zebrafish	R-metalaxyl were more toxic to aquatic organisms than rac-metalaxyl. After 24 h Na, K-ATPase enzyme activity increased by ~50% than control for 70 mg/L of R-metalaxyl but no significant values were collected at 10 mg/L. From 48 to 96, 1omg/L R-metalaxyl gradually increased. In contrast, rac-metalaxyl did not change the enzyme activity from 24 to 96 h for both concentration	[52]
Cimetidine	*Daphnia magna*: 0, 0.048, 0.24, 1.2, 6.0, and 30 mg/L *Moina macrocopa*: 0, 0.3, 3, 30, or 100 mg/L	Mortality and population growth Sex hormones and gene expression level in zebrafish	Cimetidine is not acutely toxic at levels occurring in the aquatic environment. Chronic exposure to cimetidine leads to alteration of the steroidogenesis pathway. Endocrine disruption effects were also observed in early life stage exposure	[53]
Cefadroxil and Cefadrine	*Daphnia magna*: cefadroxil (0, 5.6, 11.7, 22.2, 39.5, and 83.0 mg/L) and cefradine (0, 4.3, 9.7, 19.7, 39.0, and 80.8 mg/L). *Oryzias latipes*: cefadroxil (0, 1.0, 7.8, 84.8, 718.9, or 8883.1 mg/L) and cefradine (0, 1.0, 7.1, 73.9, 724.6, or 7758.5 mg/L).	Survival and population growth. Endocrine disruption mechanism	Exposure to these drugs caused disruptions to the functioning of the endocrine system altering gene transcription levels and sex hormone levels. Cefadroxil and cefradine impaired growth.	[3]
Four CeO2 Nanocrystalline catalyst (CuO–CeO2, CuCe20, CuCe10 and CuCe15)	*Danio rerio* and *Daphnia magna*: 1, 10, 50, 100, 250 and 500 mg/L	Hatching success and teratology effects	pure nanocrystalline CeO2 and mixed oxide CuO–CeO2 catalysts are not highly toxic as the other pollutants but still some sublethal effects of CuO–CeO2 were found	[54]
ZnO, CuO, Au, and TiO2 Nanoparticles	*Daphnia magna*: 0.5, 1, 2, 4, and 8 mg/ dm^3^ for ZnO NPs (30 nm) and CuO NPs; 1, 2, 4, 8, and 10 mg/ dm^3^ for TiO2 NPs (50 nm); and 0.05, 0.1, 0.2, 0.4, and 0.8 mg/ dm^3^ for Au NP (20 nm). *Danio rerio*: 5, 10, 15, 25, and 25 mg/dm3 for ZnO NPs (30 nm, 50 nm) and CuO NP; 100, 500, 800, and 1000 mg/ dm^3^ for TiO2 NPs (< 20 nm, 30 nm), and 0.25, 0.5, 1, 2, and 4 mg/dm^3^ for Au NP (20 nm).	Developmental effects and survival rate Biomarker analysis	Almost all four nanoparticles were toxic to both species. The number of offspring surprisingly decreased as the concentration of metal ion increased. The inhibited the CAT and SOD activities caused oxidative stress resulting physiological alteration in the early life stage	[55]
Perfluorooctane sulfonic acid (PFOS) and Perfluorooctanoic acid (PFOA)	Acute: PFOS (0, 6.25, 12.5, 25, 50, and 100 mg/L) and PFOA (0, 62.5, 125, 250, 500, and 1000 mg/L). Chronic: PFOA (0, 3.125, 6.25, 12.5, 25, and 50 mg/L) PFOS (0, 0.3125, 0.625, 1.25, 2.5, and 5 mg/L). Adult Medaka: PFOS, 0.01, 0.1, and 1 mg/L; PFOA, 0.1, 1, and 10 mg/L.	Population growth, teratological effects, Histology.	Parental exposure in Japanese medaka transferred adverse effects to offspring. The threshold of PFOS is approximately 10 times higher than that of PFOA in water *Daphnia magna* but *Moina macrocopa* was more to both compound than *Daphnia magna*.	[56]

**Table A2:** Comparison of the light and dark mean of all behavioral endpoints throughout the 80 min by using Two-way ANOVA of both zebrafish larvae and *Daphnia magna* neonates. ns, no significant difference

Behavioral Endpoints	Group Comparisons	*Danio rerio*		*Daphnia magna*	
		Significance	*p* Value	Significance	*p* Value
Distance Traveled	Control vs. 1 ppb Tebuconazole	****	<0.0001	****	<0.0001
	Control vs. 1 ppb Dimethomorph	ns	>0.9999	****	<0.0001
	Control vs. 1 ppb Difenoconazole	****	<0.0001	****	<0.0001
	Control vs. 1 ppb Imidacloprid	****	<0.0001	****	<0.0001
	Control vs. 1 ppb Tolfenpyrade	****	<0.0001	****	<0.0001
	Control vs. 1 ppb Fipronil	**	0.0031	****	<0.0001
	Control vs. 1 ppb Dinotefuran	****	<0.0001	****	<0.0001
	Control vs. 1 ppb Chlorantraniliprole	****	<0.0001	ns	0.7406
	Control vs. 1 ppb Carbarly	****	<0.0001	****	<0.0001
	Control vs. 1 ppb Cypermethrin	*	0.0135	ns	0.3779
	Control vs. 1 ppb Fenpropathrin	****	<0.0001	****	<0.0001
	Control vs. 1 ppb Acetamiprid	****	<0.0001	ns	0.1502
Burst Movement Count	Control vs. 1 ppb Tebuconazole	ns	0.5148	****	<0.0001
	Control vs. 1 ppb Dimethomorph	****	<0.0001	ns	0.2088
	Control vs. 1 ppb Difenoconazole	****	<0.0001	****	<0.0001
	Control vs. 1 ppb Imidacloprid	****	<0.0001	****	<0.0001
	Control vs. 1 ppb Tolfenpyrade	**	0.0014	****	<0.0001
	Control vs. 1 ppb Fipronil	****	<0.0001	****	<0.0001
	Control vs. 1 ppb Dinotefuran	****	<0.0001	****	<0.0001
	Control vs. 1 ppb Chlorantraniliprole	****	<0.0001	ns	0.6996
	Control vs. 1 ppb Carbarly	****	<0.0001	****	<0.0001
	Control vs. 1 ppb Cypermethrin	****	<0.0001	****	<0.0001
	Control vs. 1 ppb Fenpropathrin	****	<0.0001	****	<0.0001
	Control vs. 1 ppb Acetamiprid	****	<0.0001	****	<0.0001
Rotation Count	Control vs. 1 ppb Tebuconazole	ns	0.6257	****	<0.0001
	Control vs. 1 ppb Dimethomorph	****	<0.0001	****	<0.0001
	Control vs. 1 ppb Difenoconazole	****	<0.0001	****	<0.0001
	Control vs. 1 ppb Imidacloprid	ns	0.1705	****	<0.0001
	Control vs. 1 ppb Tolfenpyrade	ns	0.2014	****	<0.0001
	Control vs. 1 ppb Fipronil	****	<0.0001	****	<0.0001
	Control vs. 1 ppb Dinotefuran	****	<0.0001	****	<0.0001
	Control vs. 1 ppb Chlorantraniliprole	ns	0.5932	****	<0.0001
	Control vs. 1 ppb Carbarly	****	<0.0001	****	<0.0001
	Control vs. 1 ppb Cypermethrin	ns	0.1311	****	<0.0001
	Control vs. 1 ppb Fenpropathrin	****	<0.0001	****	<0.0001
	Control vs. 1 ppb Acetamiprid	****	<0.0001	****	<0.0001
